# Supplementary material for: Successional Shifts in Soil C:N:P stoichiometry and its environmental controls during forest restoration in Huize County, Qujing, Yunnan Province
Source: PLoS One. 2026 Aug 21;21(8):e0356261. doi: 10.1371/journal.pone.0356261 (PMC13496410; doi:10.1371/journal.pone.0356261)
Supplement: Supplementary file 2 [file pone.0356261.s004.zip › Data and Code/RF/Pinus yunnanensis forest/figures/importance_C.P.pdf]

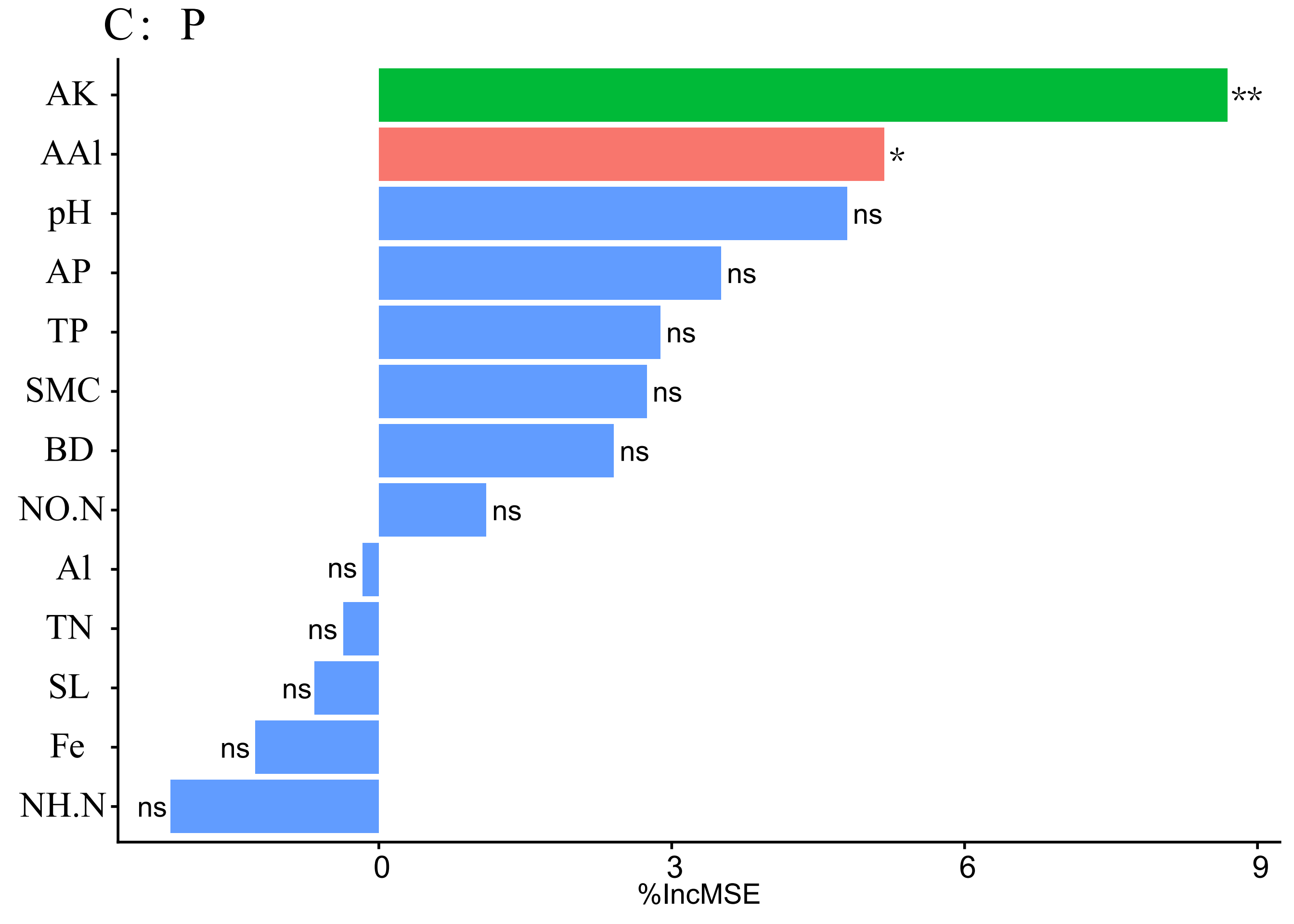
C： P
AK
AAl
pH
AP
TP
SMC
BD
NO.N
Al
TN
SL
Fe
NH.N
**
*
ns
0
3
6
9
%IncMSE
